# Functional characterization of drought-responsive modules and genes in *Oryza sativa*: a network-based approach

**DOI:** 10.3389/fgene.2015.00256

**Published:** 2015-07-30

**Authors:** Sanchari Sircar, Nita Parekh

**Affiliations:** Centre for Computational Natural Sciences and Bioinformatics, International Institute of Information TechnologyHyderabad, India

**Keywords:** functional annotation, co-expression, drought, rice, WGCNA

## Abstract

Drought is one of the major environmental stress conditions affecting the yield of rice across the globe. Unraveling the functional roles of the drought-responsive genes and their underlying molecular mechanisms will provide important leads to improve the yield of rice. Co-expression relationships derived from condition-dependent gene expression data is an effective way to identify the functional associations between genes that are part of the same biological process and may be under similar transcriptional control. For this purpose, vast amount of freely available transcriptomic data may be used. In this study, we consider gene expression data for different tissues and developmental stages in response to drought stress. We analyze the network of co-expressed genes to identify drought-responsive genes modules in a tissue and stage-specific manner based on differential expression and gene enrichment analysis. Taking cues from the systems-level behavior of these modules, we propose two approaches to identify clusters of tightly co-expressed/co-regulated genes. Using graph-centrality measures and differential gene expression, we identify biologically informative genes that lack any functional annotation. We show that using orthologous information from other plant species, the conserved co-expression patterns of the uncharacterized genes can be identified. Presence of a conserved neighborhood enables us to extrapolate functional annotation. Alternatively, we show that single ‘guide-gene’ approach can help in understanding tissue-specific transcriptional regulation of uncharacterized genes. Finally, we confirm the predicted roles of uncharacterized genes by the analysis of conserved *cis*-elements and explain the possible roles of these genes toward drought tolerance.

## Introduction

Interpretation of high-throughput data for rice toward improving its yield under varying environmental conditions is largely limited by the incomplete functional annotation of rice genes. With the seventh release of MSU Rice Genome Annotation project (http://rice.plantbiology.msu.edu/index.shtml), out of the 55,986 loci identified, around 40.9% are putative, 24.7% are expressed, 3.9% are hypothetical, 0.2% are conserved hypothetical proteins while 30.9% are transposable-element related genes. With over 50% of the genes in rice lacking annotation for biological processes (Rhee and Mutwil, 2014), there is an urgent need for annotation pipelines to be developed. Several approaches such as artificial mutagenesis followed by the analysis of phenotypic variations (Jiang and Ramachandran, 2010), systems-level annotations of genes to characterize the tissue, condition and developmental stage-specificity (Childs et al., 2011; Movahedi et al., 2011), and sequence homology-based annotation pipelines (Conesa and Götz, 2008) have been used in the functional characterization of genes. Moreover, systems level information, *viz.*, transcriptome and proteome data (Cao et al., 2012; Franceschini et al., 2013), gene regulatory information (Higo et al., 1999), metabolic and pathway level information (Dharmawardhana et al., 2013), including sequence-level information (Monaco et al., 2014) allow for an integrated and more accurate level of functional annotation.

Recently, a new approach termed *co-expression network analysis* is being used for functional annotations of genes (Childs et al., 2011; Liang et al., 2014). This is based on the observation that a coordinated participation of multiple genes is required to bring about any biological process within the cell and genes which are part of the same biological process may have similar expression profiles across different conditions. Such genes are said to be *co-expressed*. One of the most important applications of gene co-expression network analysis is to identify functional gene modules. In this study, a gene-coexpression network is constructed using weighted gene correlation network analysis (WGCNA), a package in R (Langfelder and Horvath, 2008). This method is built on the principles of graph theory where nodes correspond to genes and edges connecting them reflect correlations between gene expression profiles across samples (by typically using a weighted adjacency matrix). In general, two genes are connected if the similarity between their expression profiles is above a certain threshold, measured by Pearson correlation coefficient or other metrics. The network is then clustered into modules based on the topological overlap measure (TOM) which takes into account the correlation between two genes, as well as, the number of shared neighbors between them. This approach has been widely used on a range of systems, for e.g., co-expression network analysis of adipose genes to find genes correlated with high serum triglyceride (TG) levels (Haas et al., 2012), identify differences in transcriptome organizations between normal and autistic brain (Voineagu et al., 2011), transcriptional changes in Alzheimer’s disease and normal aging (Miller et al., 2008), biotic and abiotic stress responses using whole transcriptome sequencing in potato (Massa et al., 2013), understand seed germination in *Arabidopsis* (Bassel et al., 2011), transcriptional reprogramming in *Arabidopsis* due to mechanical wounding and insect herbivores (Appel et al., 2014), miRNA-regulated biological pathways relevant to pathogenic and symbiotic interactions in *Medicago truncatula* (Formey et al., 2014), to name a few.

Here starting with the co-expression network, we propose an approach for the characterization of functional gene modules, and functional prediction of the uncharacterized genes. We consider gene expression data of a drought tolerant rice line from different tissues and developmental stages. First, we present the analysis of the gene modules to identify tissue-specific drought-responsive gene modules based on differential expression profiles of genes comprising these modules. The modules include previously reported stress-associated genes and transcription factors along with several uncharacterized genes. Next, we identify biologically informative genes that lack functional annotation using two approaches. First, by graph-topological measures such as degree centrality and tissue-dependent differential expression of genes (*viz*., fold-change) we filter the genes. Uncharacterized genes in such a set of well-connected and differentially expressed above a certain threshold across all tissues are considered for functional annotation. Alternatively using a guide-gene approach, we select transcription factors that are up-regulated and construct a subnetwork from its strongly connected neighbors, to identify drought-stress responsive genes that lack functional annotation. Here we show that sequence-based homology search, comparison with functional networks from model plant organisms followed by motif analysis of promoters of the uncharacterized genes and their neighbors gives us an insight into the role of these genes in response to drought stress.

## Materials and Methods

### Dataset Preprocessing

In this analysis, the genome-wide temporal-spatial gene expression data of a drought tolerant rice line (GSE26280) from three tissues, leaf, root, and young panicle, at three developmental stages (tillering, panicle elongation and booting stage) exposed to drought stress, is obtained from GEO- NCBI (Wang et al., 2011). The dataset consists of 36 samples (18 drought treated and 18 control samples) consisting of 57,381 probes which are mapped to the Affymetrix annotation file for rice. Invariant set normalization, log2-transformation and filtering are performed using dChip (Li and Wong, 2001) for removing systematic variations, scaling and eliminating probes with very low intensity values respectively. The probes are filtered based on certain criteria, such as expression level be more than 20 in at least 50% of the samples and a probe is ‘present’ in at least 20% of the arrays. Around 25,804 probes satisfying the above filtering criteria are obtained. Probes that did not have any annotation or those which mapped to more than one gene are discarded. Finally, for multiple probes mapping to the same gene, the one showing a higher fold-change across all the samples is considered. After preprocessing, 18,799 unique probe-gene pairs are used for further analysis.

### Network Construction and Module Detection

The WGCNA software package in R is used to construct gene co-expression network of drought tolerant rice line from the normalized, log2-transformed expression matrix of 18,799 genes. In WGCNA, soft-thresholding is used for finding similarity relationships between gene-pairs. This is carried out by computing the unsigned Pearson’s correlation matrix and then scaling it by power β=8 (soft-threshold, based on approximate scale free-topology criterion). Subsequently, the function *block-wiseModules* is used for hierarchical clustering of genes using Dynamic Tree Cut approach (Langfelder et al., 2008) with maximum block size of 8000, minimum module size of 200, a “cut height” of 0.995 and “deep split” = 2. These results in 16 co-expressed modules ranging from 3798 (turquoise) to 296 (lightcyan) genes, with 360 genes left unclustered (grouped as gray module).

### Network Validation

#### Statistical Significance of Network Modules

To test the robustness of co-expressed modules obtained in our network, re-sampling of the dataset is carried out to estimate the module quality statistics. Using *modulePreservation* function in WGCNA (Langfelder et al., 2011), 200 permutations are performed and log *p*-values and *Z*-scores for various network quality statistics such as density, module membership, connectivity, etc. for each module are computed which are summarized as *psummary* and *Zsummary* (see Supplementary Table S1). The *Z*-score provides evidence that a module is preserved more significantly than a random sample of all network genes, while *p*-value gives the probability of seeing the module quality statistic in a random sample of genes of the same size. Here we observe that the *psummary* value is very low (∼0.0) and *Zsummary* > 10, thus providing strong evidence of network connectivity preservation and robustness of all the co-expressed modules.

#### Biological Relevance of Network Modules

The tissue-specificity of the modules based on the percentage of differentially expressed genes (DEGs) and their functions define the relevance of these modules toward various biological processes which are switched on, off, or remain unaffected in response to drought. For this purpose, differentially regulated genes are identified in a tissue and stage-specific manner at fourfold change with *p*-value ≤0.05 using dChip. We observe that ∼17% genes are differentially expressed in at least one of the tissues. Gene ontology and enrichment analysis of each of the modules is performed using agriGO (Du et al., 2010), RiceNetDB (Liu et al., 2013a), and RGAP (Kawahara et al., 2013). The RGAP database is also used for comparison of co-expression profiles and gene-pair associations with other rice datasets. The modules are visualized using Cytoscape (Shannon et al., 2003).

#### Alternative Network Inference Methods

For assessing the biological significance of gene-pair associations in the conserved gene clusters used for functional extrapolation, we constructed four co-expression networks based on different methods. Two methods of network construction used for the analysis are: correlation based and context likelihood of relatedness (CLR) based methods (Faith et al., 2007). For correlation based networks, two different association rules, *viz*., Pearson correlation and Spearman rank correlation were used, and for the CLR-based networks, two reverse engineering methods, *viz*., mutual information (MI) and maximal information coefficient (MIC) were used, (Reshef et al., 2011). The alternate network construction was done using DeGNServer (Li et al., 2013) and Markov Cluster Algorithm (MCL; Enright et al., 2002) was used for the clustering of co-expressed genes in these networks. The parameters used for construction and other details of these four networks are given in Supplementary Table S2.

## Results

### Identification of Drought-Responsive Modules: Tissue Specificity

**FIGURE 1:**
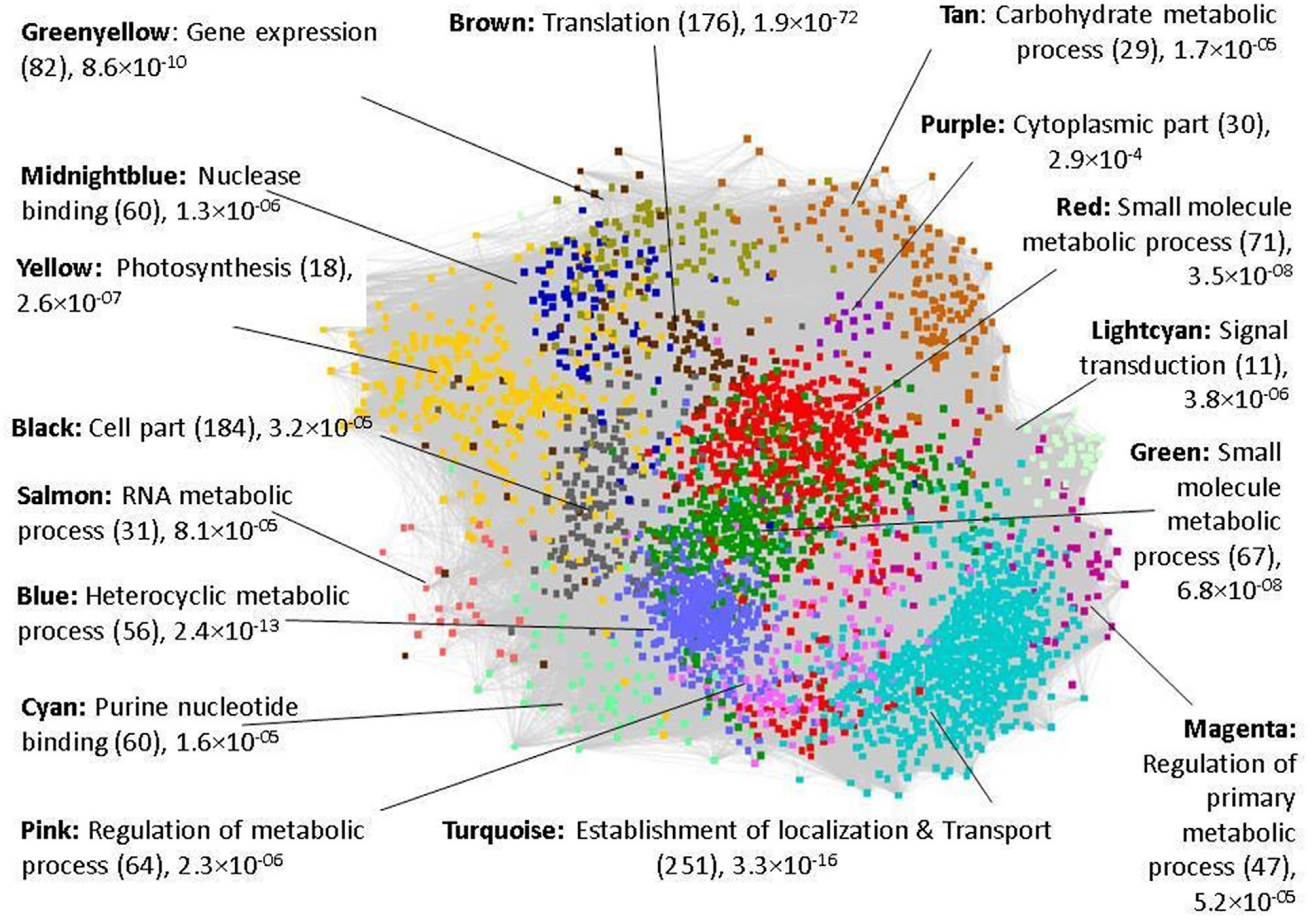
**Functional modules with GO enriched terms.** The number in bracket corresponds to genes annotated for the top GO term along with the *p*-value. Only the differentially expressed genes (DEGs) are highlighted for ease of visualization in each module, depicted using a representative color code.

**FIGURE 2:**
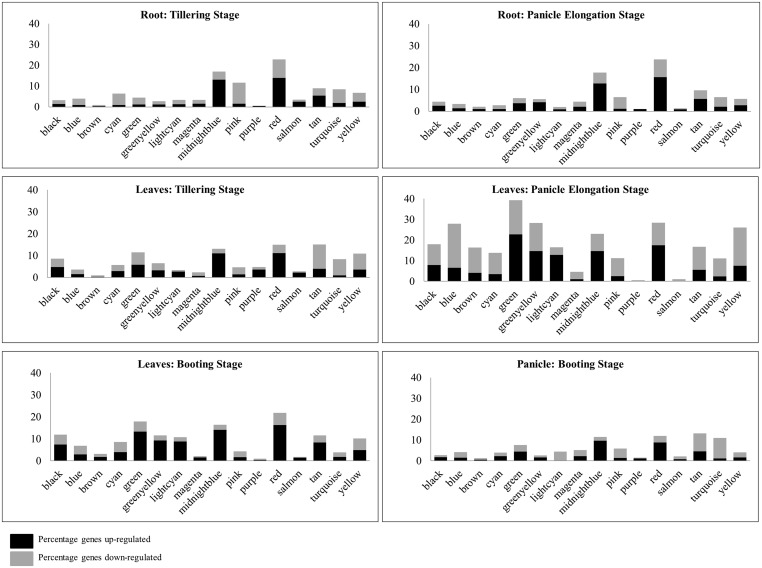
**Percentage of DEGs across tissues and developmental stages.** The distribution of up- and down-regulated genes across modules in various tissues may help in identifying important processes being switched on or off in response to drought.

The 16 co-expressed gene modules obtained using WGCNA are analyzed to get an insight into the function of these co-expressed gene clusters. The GO enrichment analysis of the modules is performed by submitting the complete gene list of each module to agriGO and the statistical significance is determined using *Fisher’s exact test* at *p*-value <0.05. In **Figure 1**, the general function of the sixteen co-expressed modules (represented in different colors) is given based on the most enriched GO term and *p*-value. Since the general function of the modules do not give any indication of the core set of genes/modules that may respond to drought stress, we next analyzed the differential expression of genes in a tissue-specific manner. The percentage of DEGs in each module at various developmental stages in the three tissues is identified and depicted in **Figure 2**. It may be noted that the red and midnightblue modules exhibit a high percentage of DEGs ubiquitously across all the three tissues and developmental stages. That is, these two modules comprise of important drought-responsive genes and we discuss their analysis in detail below. On the other hand, purple, salmon and magenta modules show very low percentage of DEGs across various tissues indicating their negligible role in drought response. In the panicle elongation stage in leaves, almost all the modules exhibit a high percentage of DEGs (∼18.5%) suggesting it to be the most important drought affected stage in plant. The analysis of DEGs would give an insight into various drought-responsive molecular processes activated during this stage in rice. For e.g., in the brown module, a high percentage of genes down-regulated in the panicle elongation stage in leaves, are associated with gene expression, translation and protein metabolic processes. This may be because of the various high energy requiring processes shut down during drought in leaves. Similarly, pink module exhibits a significant number of down-regulated genes in root tillering stage and in leaves panicle elongation stage. Some of these genes are involved in oxidoreductase activity, some as auxin-responsive genes [suggesting a decrease in lateral root development (Casimiro et al., 2001)] and some involved in the biosynthesis of secondary cell wall. In the tan module, genes up-regulated in both the stages in root are identified to be involved in ubiquitin mediated proteolysis, plant hormone signal transductions (phosphatases) and polymeric compound degradation including starch (chitinase and β-amylases). These have been implicated in remobilization of complex polymers to provide soluble sugars during stress conditions (Seiler et al., 2011).

In leaves, the tan module has a higher percentage of down-regulated genes in tillering and panicle elongation stage compared to the booting stage. Some of these down-regulated genes are associated to primary and secondary cell wall biosynthesis and signal transduction (ras-related proteins). In panicle elongation stage, all the modules, except purple and salmon, exhibit a large percentage of DEGs. Of these, green, red, midnightblue, greenyellow, and lightcyan modules show a high percentage of up-regulated genes, while the remaining modules exhibit a higher percentage of down-regulated genes. This stage being a reproductive stage, the plant is particularly sensitive to water requirements and certain processes are preferentially activated or shut down as apparent from the subsequent functional analysis. In the booting stage, when the panicle has already grown to a certain height, the percentage of down-regulated genes is observed to be reduced in most of the modules. Red and midnightblue are the most important modules in terms of DEGs in both tillering and panicle elongation stages in root, while the pink module exhibit higher number of DEGs only in the tillering stage. In the panicle booting stage, the percentage of DEGs is much lower compared to all other tissues. About 10–12% of genes are differentially expressed in four modules, namely, red and midnightblue (mostly up-regulated) and tan and turquoise (mostly down-regulated). Thus, we see that by a systematic analysis of DEGs module-wise, we can identify tissue-specific functional roles of the modules in response to environmental stress. The analysis of clusters or subnetworks of DEGs in these modules may provide insight into the molecular processes activated in various tissues/stages.

Below we discuss a detailed analysis of some of these modules to understand the underlying mechanisms in various tissues at different development stages in response to drought. In particular, we discuss the analysis of red and midnightblue modules that show a significant percentage of DEGs across all the tissues, green, yellow, and blue modules that show differential expression in the panicle elongation stage in leaves, and turquoise module for the panicle booting stage. We also use graph-based approaches to identify important genes in the drought-induced modules, red, midnightblue, and green, that lacks any functional annotation. Two alternative approaches have been proposed for the function prediction of these uncharacterized genes.

### Essential Drought-Responsive Modules

As is evident from **Figure 2**, a significant fraction of genes in red and midnightblue modules are differentially expressed (mostly up-regulated) ubiquitously in all the three tissues and developmental stages. In fact, percentage of DEGs common between root and leaf tissues is ∼40% in the red module and ∼47% in the midnightblue modules respectively. This suggests that the two modules comprise a core set of genes that are involved in drought-responsive processes at various development stages in the plant. Below we present a detailed analysis of these two modules to understand the functional role of the DEGs.

#### Analysis of Red Module

The red module consists of 1344 genes, a large fraction of which are involved in metabolism (∼45%), ∼18% in response to stimulus and ∼9% in abiotic stress (data obtained from RiceNetDB). We observe that ∼34.3% (461) of the genes exhibit fourfold or higher differential expression in at least one of the tissues (14.6, 16, and 9% up-regulated and 8.4, 7, and 3% down-regulated in root, leaf and panicle respectively). Of these 95 DEGs (∼21%) are annotated as “expressed proteins,” having transcript-level evidence but lack specific GO annotation for biological processes. We also observe that 57 genes are differentially expressed across all tissues and stages at fourfold or more (56 up-regulated and 1 down-regulated), suggesting their ubiquitous role in drought stress response. Phospholipase D (PLDα4) gene is down-regulated across all the stages. PLDs are known to have a role in lipid metabolism, growth and development and PLDα4 is reported to be suppressed by most plant hormones including abscisic acid (ABA; Li et al., 2007). Functional analysis of the 56 up-regulated genes in agriGO and RGAP suggest their association with reproduction and post-embryonic development (LEAs, seed maturation proteins, CBS domain containing membrane protein, embryonic protein DC-8), stress responsive proteins (e.g., OsRCI2-5 and OsRCI2-7, dehydrins), and metabolic processes (e.g., phosphoglycerate mutase, dehydrogenase E1 component domain protein, transketolase-chloroplast precursor, glutathione *S*-transferase, cytokinin-*O*-glucosyltransferase 2, etc.).

##### Degree centrality analysis

To analyze if the DEGs are also well-connected with other genes, we carried out centrality-based analysis of top 20% (269) high-degree (‘hub’) genes in the red module. Numerous studies have shown that genes/proteins with high degree tend to be essential for the organisms (Jeong et al., 2001; Barabási and Oltvai, 2004). We observe that out of 269 hub genes, about 145 genes are differentially expressed across at least one of the tissues. Interestingly, 52 of the 56 up-regulated DEGs in the red module are also the hub genes. Of these 13 are uncharacterized “expressed proteins.” These 13 genes that are well-connected and up-regulated across all tissues and stages are thus ideal candidates for further functional analysis.

**FIGURE 3:**
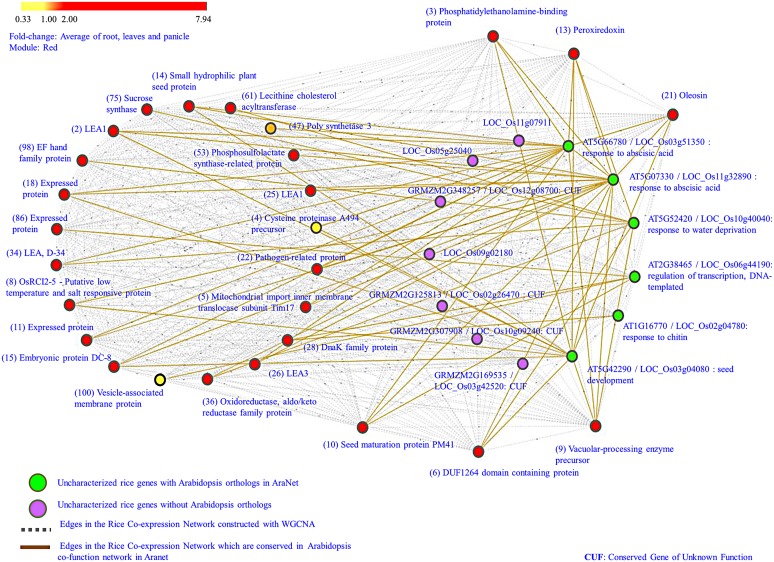
**Uncharacterized genes in red module.** Genes that are high degree and up-regulated across all developmental stages and tissues are depicted in ‘green’ color (having *Arabidopsis* orthologs) and ‘purple’ color (having no *Arabidopsis* orthologs). Their neighbors, identified as orthologs in the co-function network in AraNet are colored according to the average fold-change in drought samples. The edges in ‘brown’ denote conserved co-expressed links among genes in rice and *Arabidopsis*, while the ‘gray edges correspond to co-expressed links in red module. The numbers in the bracket indicate the rank of the neighbors (based on edge weight) in AraNet.

As a first step toward understanding the functional role of these 13 uncharacterized genes, homology-search for orthologs in other plant species was carried out using AraNet and RGAP database. AraNet is a genome-wide, condition-independent functional network of *Arabidopsis* genes reconstructed by integrating functional genomics, proteomics, and comparative genomics datasets. The functional linkages among gene-pairs are weighted by the log likelihood of the linked genes to participate in the same biological processes (inferred from direct assays, protein–protein interactions, sequence/structure similarity, literature mining, etc.). The Rice Genome Annotation Database (RGAP; Kawahara et al., 2013) is another important resource that provides sequence and annotation data for the rice genome including information about rice orthologous groups in *Arabidopsis*, maize, grapevine, poplar, etc. It also provides co-expression patterns between gene-pairs from 15 different rice gene expression experiments. On searching in AraNet, orthologs for only 6 of the 13 uncharacterized rice genes were found; however, these orthologs also lacked any specific functional annotation for biological processes. Next, from the co-function network in AraNet, we extracted top 100 neighbors (ranked by total edge weight score) of the six *Arabidopsis* orthologs and mapped them on to our rice co-expression network. Rice orthologs for 82 of these 100 *Arabidopsis* neighbors were identified in our co-expression network. As expected, majority of the high-ranked neighbors of *Arabidopsis* orthologs mapped to the red module (∼34%), with a smaller fraction mapping to turquoise (∼21%) and blue (∼ 8%) modules. We observe that for the 6 uncharacterized genes, 27 neighbors, and 60 edges in this cluster are conserved between *Arabidopsis* and red module in our network. In **Figure 3** the conserved edges are depicted in brown color between the six uncharacterized genes shown in green and 27 conserved network neighbors mapped on to the red module. It is observed that these 27 genes are mostly up-regulated, especially in root and leaf, and are well-connected in the network as shown by gray edges in **Figure 3**. Majority of these genes are involved in seed development (embryonic protein DC-8, LEAs, seed maturation protein PM41, small hydrophilic plant seed protein) and in biotic and abiotic stress response (pathogen-related protein, OsRCI2-5, DnaK family protein, etc.). AraNet prioritizes the putative ontology of a gene based on the most enriched ontologies among its neighbors. Extrapolating the annotation from the *Arabidopsis* orthologs in AraNet to the six uncharacterized rice genes, we assign the following GO term to these genes: “regulation of transcription,” “response to ABA,” “seed development,” “response to water deprivation” and “response to chitin,” as shown in **Table 1**. For the remaining seven uncharacterized genes, ortholog search in other plant species resulted in orthologs for four genes in maize, but again with no specific functional annotation.

**Table 1:** Functional annotation of uncharacterized genes in red, midnightblue, and green modules.

Uncharacterized “expressed proteins”	Homology-based annotation	Annotation from conserved co-expressed neighbors (AraNet and RGAP)	Annotation from promoter analysis (MEME, PLACE, STAMP)
**RED**			
LOC_Os06g44190	*Arabidopsis*: AT2G38465	Regulation of transcription	ABRE, AGTACSAO, C2GMAUX28
LOC_Os03g51350	*Arabidopsis*: AT5G66780	Response to ABA	ABRE, AGTACSAO, C2GMAUX28
LOC_Os11g32890	*Arabidopsis*: AT5G07330	Response to ABA	ABRE, AGTACSAO, C2GMAUX28
LOC_Os03g04080	*Arabidopsis*: AT5G42290	Seed development	ABRE, AGTACSAO, C2GMAUX28
LOC_Os10g40040	*Arabidopsis*: AT5G52420	Response to water deprivation	ABRE, AGTACSAO, C2GMAUX28
LOC_Os02g04780	*Arabidopsis*: AT1G16770	Response to chitin	ABRE, AGTACSAO, C2GMAUX28
LOC_Os03g42520	Maize: GRMZM2G169535	Multicellular organismal development	ABRE, AGTACSAO, C2GMAUX28
LOC_Os12g08700	Maize: GRMZM2G348257	–	ABRE, AGTACSAO, C2GMAUX28
LOC_Os02g26470	Maize: GRMZM2G125813	–	ABRE, AGTACSAO, C2GMAUX28
LOC_Os10g09240	Maize: GRMZM2G307908	–	ABRE, AGTACSAO, C2GMAUX28
LOC_Os09g02180	–	–	ABRE, AGTACSAO, C2GMAUX28
LOC_Os11g07911	–	–	ABRE, AGTACSAO, C2GMAUX28
LOC_Os05g25040	–	–	ABRE, AGTACSAO, C2GMAUX28
**MIDNIGHTBLUE**			
LOC_Os01g73110	*Arabidopsis*: AT5G45310	Floral organ abscission, response to ABA	ABRE
LOC_Os10g36180	*Arabidopsis*: AT5G52300 (RD29B)	Response to abiotic stimulus, endogenous stimulus and ABA, signal transduction, response to water deprivation.	ABRE
LOC_Os08g01370	*Arabidopsis*: AT3G12960 (seed maturation protein)	Zinc ion transport, protein folding	ABRE
**GREEN**			
LOC_Os03g60520	Maize: GRMZM2G017682 (Phosphatidylinositol *n*-acetylglucosaminyltransferase subunit p)	–	ABRE, MYB, WRKY71, CACTFTPPCA1
LOC_Os03g19580	*Arabidopsis*: AT3G62370 (heme binding)	Response to water deprivation, metabolic process	ABRE, MYB, WRKY71, CACTFTPPCA1
LOC_Os02g37180	Maize: GRMZM2G083855 (metal binding protein)	–	ABRE, MYB, WRKY71, CACTFTPPCA1
LOC_Os08g34800	*Arabidopsis*: AT5G57510	Response to chitin	ABRE, MYB, WRKY71, CACTFTPPCA1
LOC_Os12g05690	*Arabidopsis*: AT3G10120	Protein modification process, biosynthetic process	ABRE, MYB, WRKY71, CACTFTPPCA1
LOC_Os02g53410	Maize: GRMZM2G139293 (conserved gene, unknown function)	–	ABRE, MYB, WRKY71, CACTFTPPCA1
LOC_Os07g33280	–	–	ABRE, MYB, WRKY71, CACTFTPPCA1
LOC_Os12g25090	–	–	ABRE, MYB, WRKY71, CACTFTPPCA1
LOC_Os11g10470	–		ABRE, MYB, WRKY71, CACTFTPPCA1
LOC_Os06g05410	–	–	ABRE, MYB, WRKY71, CACTFTPPCA1

A search of these 13 uncharacterized genes and 27 conserved neighbors was carried out in RGAP database to further confirm their coexpression. It was observed that 10 out of 13 uncharacterized genes, and 11 out of 27 annotated genes are co-expressed and belong to the same module (turquoise) in the experiment GSE6901 (GSE6901- 7-days-old rice seedlings grown in the presence of light and under control and stress conditions: drought, cold, and salinity). This includes five of the remaining seven uncharacterized genes with no *Arabidopsis* orthologs. (The co-expression profiles are given in Supplementary Figure S1). The GSE6901-turquoise module is shown to be associated with genes differentially expressed due to drought and salt stress by Childs et al. (2011). The conservation of co-expression profiles of 21 out of 40 genes in an independent experimental study further provides biological significance to the associations between these genes.

Further evidence toward biological relevance is provided by analyzing these associations in four different network constructions. For the Spearman’s rank correlation network and the MI-based CLR network, we observe that all the 40 genes are clustered in the same module and 329 and 293 edges are conserved respectively between the 13 uncharacterized and 27 characterized genes. For the Pearson correlation network and the MIC-based CLR network, 37 genes are clustered together in the same module with 297 and 140 edges between them respectively (details given in Supplementary Table S3). Thus, based on the inference from alternate methods of networks construction, conserved neighborhood from AraNet and conserved co-expression profiles in independent experimental study in RGAP, we conclude the relatedness between these 40 genes under drought stress.

##### Analysis of *cis*-regulatory elements

Co-expressed genes that are densely connected to each other in a functional module are likely to share similar short responsive elements. From the annotation of the network neighbors of the uncharacterized genes (**Figure 3**), we observe that these are LEAs and other stress-responsive genes with a known role in ABA response. So it is highly likely that the uncharacterized genes may also have a role in the ABA signaling pathway. ABA is a regulatory molecule involved in drought stress tolerance and its main function is to regulate osmotic stress tolerance via cellular dehydration tolerance genes. ABA-inducible genes have the ABA-responsive element (ABRE) in their promoters (Debnath et al., 2011). To check for the presence of ABRE in the uncharacterized genes, 1 kb upstream region of 40 gene sequences (13 uncharacterized genes and 27 conserved network neighbors from **Figure 3**) are analyzed using the Plant *Cis*-acting Regulatory DNA Elements (PLACE) database (Higo, 1998). All the 40 genes had a number of (ABREs in the promoter region, e.g., ABRELATERD1 (Simpson et al., 2003; Nakashima et al., 2006), ABRERATCAL (Kaplan et al., 2006), ABREATCONSENSUS (Choi et al., 2000), etc. In order to see if these genes share any other regulatory motifs, the 1 kb upstream region of these gene sequences were analyzed using the motif discovery tool, MEME (Bailey et al., 2009). The predicted motifs were then searched in the STAMP server (Mahony and Benos, 2007), which performs motif alignments against various motif databases. Almost all the sequences were enriched for the AGTACSAO element which is probably linked to auxin (Kisu et al., 1998) and C2GMAUX28 also associated with auxin-responsive genes (Nagao et al., 1993). The presence of both ABRE and auxin-associated *cis*-elements suggest a possible link between these two phytohormones. The interdependency between these two hormones has been recently studied by Liu et al. (2013b). They reported that a cross-talk exists between auxin action in seed dormancy and ABA signaling pathway and showed that auxin acts upstream of ABI3 (major regulator of seed dormancy) by recruiting the ARF 10 and 16 to control the expression of ABI3 during seed germination.

#### Analysis of the Midnightblue Module

It is one of the smaller modules with only 383 genes. Functional enrichment analysis of this module in RiceNetDB indicate that about 29% of the genes belong to primary metabolism, 16% to response to stimulus, and 14% to nucleobase, nucleoside, nucleotide, and nucleic acid metabolism. Of these 98 genes (∼26%) are differentially expressed at fourfold in at least one of the tissues. In all the three tissues, percentage of up-regulated genes is higher (∼12.8, 12.5, and 9.7% in root, leaf and panicle respectively) compared to that of down-regulated genes (∼4.4, 3.9, and 1.8%). About ∼18% of the DEGs are ‘expressed proteins’ with no functional annotation. The GO analysis revealed that this module contains a number of proteins involved in nucleotide binding, specifically ATP binding. Some of the up-regulated genes of this module involved in ATP binding activities are AAA-type ATPase family protein, ABC transporter-ATP-binding protein, plant PDR-ABC transporter associated protein, NBS-LRR disease resistance protein, plasma membrane ATPase, etc. Apart from these, genes involved in RNA biosynthetic process such as AP2/EREBP transcription factors (*LOC_Os08g36920* and *LOC_Os06g07030*), bZIP transcription factor (*LOC_Os01g64730*), translation initiation factor SUI1, Homeobox domain containing protein (*LOC_Os01g19694*) are observed to be up-regulated. The AP2/EREBP (APETALA2/ethylene-responsive element-binding protein) is a large family of TF genes in the plant kingdom involved in a myriad of functions such as seed development, organ development, response to biotic and abiotic stress, etc. (Sharoni et al., 2011). The bZIP transcription factors belong to a large family of regulatory proteins involved in seed development and maturation, and stress response primarily through ABA-dependent signaling pathways (Jakoby et al., 2002; Xu et al., 2012).

##### Degree Centrality Analysis

From topological network analysis, we observe that out of top 20% of the high-degree genes, ∼56% are up-regulated in at least one of the tissues. Analysis of the down-regulated genes show that the invertase/pectin methylesterase inhibitor family protein (*LOC_Os10g36500*) is down-regulated especially in root and leaf and has a role in basal disease resistance and tolerance to oxidative stress as shown in a recent meta-analytic study on rice biotic and abiotic stress conditions (Shaik and Ramakrishna, 2013). The gene, lachrymatory factor synthase (*LOC_Os01g10210*), found to be significantly down-regulated across all the tissues, has its ortholog in onion reported to be involved in secondary metabolism (Jones et al., 2004). We observe that 18 genes are up-regulated at fourfold across all tissues and stages. These genes are involved in nucleobase-containing compound metabolic processes including those associated with ATP (AAA-type ATPase family protein, ATP-dependent protease La, ABC transporter-ATP binding protein) as well as transcription factors like bZIP (*LOC_Os01g64730*), HSF (*LOC_Os06g35960*), and HB (*LOC_Os01g19694*), genes involved in various responses to stimulus such as stem-specific protein TSJT1, protein phosphatase 2C, protein disulfide isomerase (involved in cell growth and differentiation), cytochrome c oxidase subunit, etc. Apart from these, three “expressed proteins” are up-regulated across all the tissues and the developmental stages. From homology-based analysis of the three uncharacterized genes in AraNet and RGAP database, we found that two of these have characterized orthologs: ortholog of *LOC_Os10g36180* is “responsive to dessication-29B” (RD29B) and is involved in ABA signaling pathway, leaf senescence, response to salt, cold and water deprivation and the ortholog of *LOC_Os08g01370* is a seed maturation protein in *Arabidopsis*. The analysis of the third uncharacterized gene (*LOC_Os01g73110*) in AraNet and RGAP suggests its role in floral organ abscission and response to ABA. Identification of *cis*-regulatory elements in PLACE database indicates that all the three uncharacterized genes have ABREs in their promoter regions suggesting that these are activated in an ABA-dependent manner. The promoter analysis is in accordance with the predicted annotation and the results are summarized in **Table 1**.

### Leaves-Specific Module

In the tillering stage in leaves, tan, green and yellow modules show a significant fraction of DEGs apart from red and midnightblue modules. While in the panicle elongation stage, all the modules except purple, salmon and magenta show a very high fraction of DEGs and a similar trend (but with a lower percentage) is observed in the booting stage in leaves. The onset of reproductive stage is marked by the elongation of panicle and by booting stage, the panicle is completely developed. The morphology of the panicle is one of the main determinants of rice yield (Furutani et al., 2006). Previous studies have shown that a large number of genes, especially transcription factors, are differentially expressed during the early stages of panicle development due to the series of rapid morphological changes occurring in the plant (Zhang et al., 2005; Furutani et al., 2006). In our analysis we observe that the modules exhibiting a high percentage of DEGs specific to the panicle elongation stage in leaves are: green (∼23% up-regulated and 16.6% down-regulated), greenyellow (∼15% up-regulated and 13.7% down-regulated), yellow (∼7.5% up-regulated and 18.6%) and blue (∼6.6% up-regulated and ∼21.4% down-regulated) modules. While the DEGs in greenyellow module did not show a significant GO enrichment, down-regulated genes in the blue module were found to be involved in photosynthesis and tetrapyrrole (chlorophyll) biosynthetic process indicating that photosynthesis is inhibited during drought. A similar trend is observed in the yellow module with genes associated with photosynthesis, localization and transport being down-regulated.

#### Analysis of Green Module

The green module exhibits a high percentage of DEGs in leaves (∼14.3% up-regulated and 8% down-regulated) compared to roots and panicle. This module is characterized by the presence of many differentially expressed transcription factors probably due to the transition from a vegetative stage (tillering) to reproductive stage (panicle elongation), as the plant balances between countering drought and sustaining the growth of the plant. About 57 genes of this module are observed to be up-regulated in all the stages of leaves. These include genes involved in response to stimulus as well as a number of transcription factors such as MYB, AP2-EREBP, WRKY (WRKY72 and WRKY55), bZIP, U-box domain containing protein, WIP3 -wound-induced protein precursor, thaumatin, ZOS12-09 – C2H2 zinc finger protein, etc. About 31 genes of this module are observed to be down-regulated across all stages in leaves. These include genes involved in metabolism (dehydrogenase, OsSub12- Putative Subtilisin homolog, phosphoribosyltransferase, ZOS7-13 – C2H2 zinc finger protein, chlorophyll *a/b* binding protein, ent-kaurene synthase, chloroplast precursors, omega-3 fatty acid desaturase, chloroplast precursor, etc.).

##### Analysis of transcription factor: OsMYB2

**FIGURE 4:**
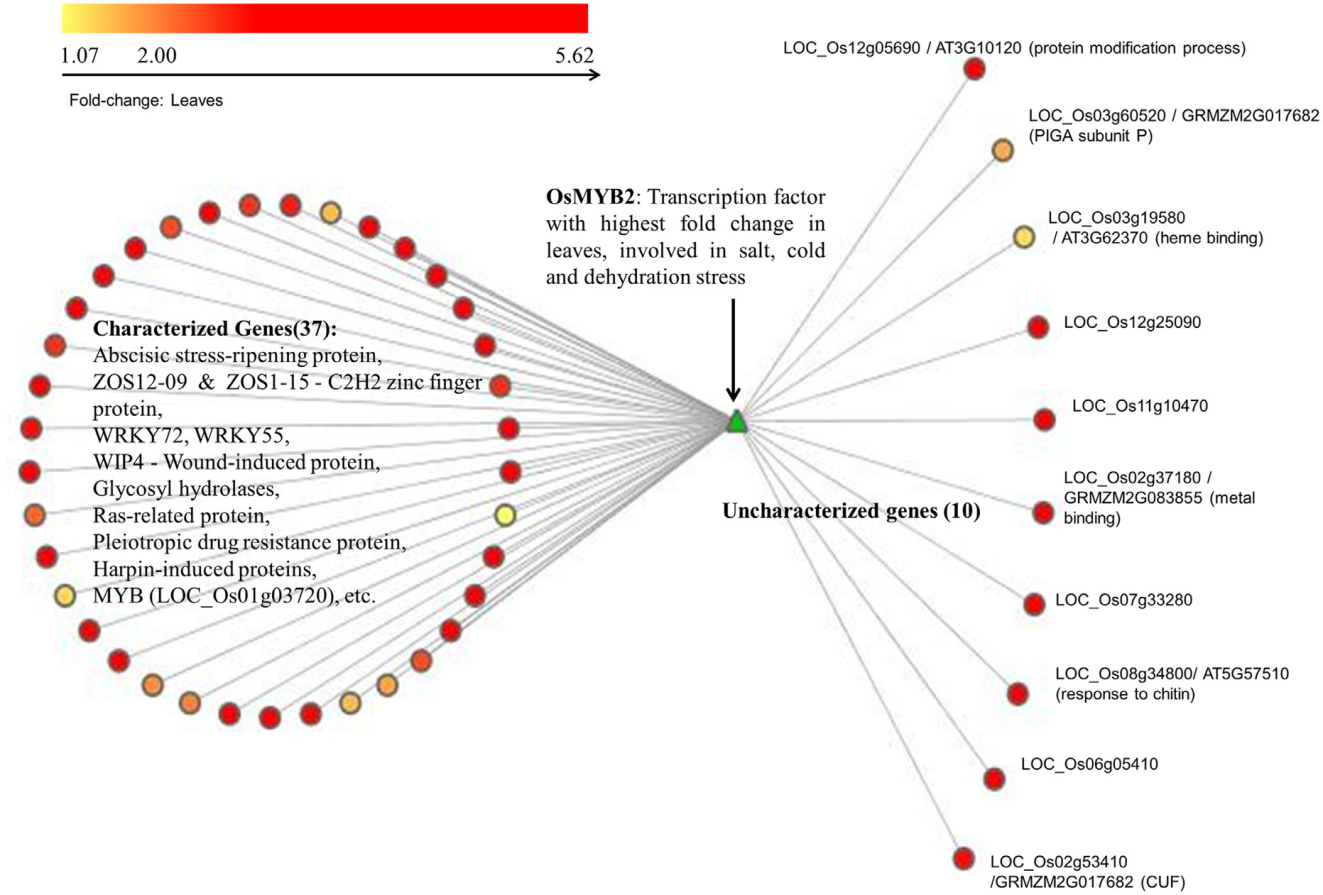
**Uncharacterized neighbors of OsMYB2 in green module.** The R2R3 transcription factor, OsMYB2 is depicted in green. Its first neighbors are depicted according to the fold change observed in leaves. Among these, 37 are characterized and 10 are uncharacterized proteins.

The green module has a number of transcription factors that are differentially expressed in leaves across various stages. Here we present an alternative approach for the functional annotation of genes that are tightly coupled, differentially expressed and are likely to be co-regulated by a transcription factor. As a representative example, we consider R2R3-MYB transcription factor OsMYB2 (*LOC_Os07g48870*) as the ‘guide-gene,’ which is known to play a role in salt, cold and dehydration stress (Yang et al., 2012). The top 5% first-degree neighbors (69) of this transcription factor, OsMYB2, are considered for further analysis. We observe that 47 of these first-neighbors of MYB are up-regulated at twofold change or higher, 15 are down-regulated, and seven genes did not show any significant fold-change. The GO analysis of the 47 up-regulated genes indicate that 37 of these are involved in stress response (such as ABA stress-ripening protein, pleiotropic drug resistance protein, receptor-like protein kinase HAIKU2 precursor, harpin-induced proteins, white-brown complex homolog protein 11, oxidoreductase, aldo/keto reductase family protein, hypoxia-responsive family protein, glycosyl hydrolase, WIP4 – wound-induced protein precursor, etc.) and 10 are uncharacterized genes as shown in **Figure 4**. Apart from these, stress-induced transcription factors such as ZOS12-09-C2H2 zinc finger protein, WRKY72, WRKY55 and MYB family transcription factor (*LOC_Os01g03720*) are also included suggesting that OsMYB2, which exhibits a higher fold-change compared to other differentially expressed transcription factors, may be functioning as a master-regulator in response to drought in leaves. Of the 10 uncharacterized genes, only three have characterized orthologs in RGAP, a PIGA (Phosphatidylinositol *n*-acetylglucosaminyltransferase) subunit *p* involved in lipid metabolism (LOC_Os03g60520), a heme binding (LOC_Os03g19580) and a metal binding protein probably involved in chlorophyll binding (LOC_Os02g37180). The functional extrapolation for the three uncharacterized genes from AraNet is given in **Table 1**. Since no ortholog of OsMYB2 is known in *Arabidopsis*, we searched these 47 genes in the RGAP database. We found that 26 out of 37 annotated genes and 7 out of 10 uncharacterized genes are reported to be positively correlated with OsMYB2 in at least one of the experiments in RGAP (GSE17245, GSE6901, GSE6893, and GSE19024), providing evidence for the association between these genes with OsMYB2 (Childs et al., 2011). The association between the 48 genes (OsMYB2 and its 47 neighbors) was examined in four alternate network constructions as well. We observed that in Pearson and Spearman rank correlation-based networks, 48 and 47 genes are clustered together and 47 and 46 edges respectively with OsMYB2 in the two networks. Similarly, in the case of MI and MIC based CLR networks, 48 and 45 genes are clustered together in the same module along with 46 and 32 edges with OsMYB2 respectively (details given in Supplementary Table S3). The conserved associations in independent experiments in RGAP and in four alternate inference methods, suggests that OsMYB2 may indeed be regulating these 47 genes. Thus, in this case with no known ortholog in the model organism, the single-guide gene approach provides a reliable approach for function annotation. For further confirmation and functional annotation, promoter analysis of these 47 genes for the presence of MYB motif is carried out.

To predict the functional role of remaining seven uncharacterized genes, 1 kb upstream sequences of the up-regulated neighbors (47 genes including 10 uncharacterized genes) of the transcription factor OsMYB2 were analyzed using MEME. The predicted *cis*-regulatory elements from MEME were filtered based on the frequency of occurrence in the promoter regions of the 47 genes and searched against databases of known motifs using STAMP. The motif analysis suggests that these genes may be possible targets of the AtSPL8 transcription families [involved in sporogenesis in anthers and ovules (Unte et al., 2003)], AtMYB15 [involved in enhanced sensitivity to ABA and improved drought tolerance (Ding et al., 2009)] and ABI4_2 (involved in seed germination, plastid-to-nucleus signaling, sugar signaling (Bossi et al., 2009; Zhang et al., 2013). The 1 kb upstream sequences of the 10 uncharacterized genes were individually searched against the PLACE database. In all the 10 sequences, motifs for ABRE and MYB binding sites were observed. The role of ABA signaling pathway in drought response is well known and the co-presence of MYB sites suggest its role in ABA signaling pathway. Two other interesting motifs observed were WRKY71 and CACTFTPPCA1. It is known that the gene OsWRK71 is a transcriptional repressor of gibberellins (GA) signaling. Recent studies indicate that inhibition of GA-signaling promotes drought tolerance by forming smaller stomatal pores, and reducing leaf desiccation (Nir et al., 2014; Zawaski and Busov, 2014). The CACTFTPPCA1 motif occurred in high frequency in all the 10 sequences and the ‘CACT’ is a key component for mesophyll (leaves)-specific gene expression in the C4 plants (Gowik et al., 2004). Thus, the presence of these regulatory elements further confirm the role of these uncharacterized genes as specific to leaves and probably involved in desiccation tolerance. The results of the analysis are summarized in **Table 1**.

### Panicle-Specific Module

In panicle booting stage, we observe very few DEGs compared to other tissues. About 10% of the DEGs belong to the four modules, red, midnightblue, tan and turquoise. The turquoise module has a large fraction of down-regulated genes in this stage compared to other tissues. Based on the DEGs, we observe a number of processes to be switched-off in this module. For example, genes involved in carbohydrate metabolic processes including several “glycosyl hydrolases” and “cellulose synthases” which are involved in cellulose biosynthesis are down-regulated. Genes involved in microtubule-based movements (having kinesin motor domains) are down-regulated suggesting that processes associated with cell cycle, cell elongation and tissue expansion are probably affected due to drought in panicle. A number of peroxide precursors involved in oxidoreductase activities like ROS scavenging activities are down-regulated. Another interesting cluster of genes involved in auxin response (OsSAUR57, OsSAUR33, OsIAA31, etc.) are also down-regulated in the panicle. As auxin-responsive pathways are commonly associated with differentiation and development (Zhao, 2010) and their repression have been linked to plant defense responses (Wang et al., 2007), panicle growth and development is probably limited in this stage in response to drought. Few genes that are up-regulated at greater than twofold change in this tissue include transcription factors AP2-EREBP (*LOC_Os09g11480*, *LOC_Os05g29810*, and *LOC_Os01g49830*) and genes involved in stress response (FAD binding domain of DNA photolyase domain containing protein, universal stress protein domain containing proteins, uvrB/uvrC motif family protein, dehydrins, etc.). The AP2-EREBP transcription factor family is specific to plants and has been shown to be involved in various developmental processes, pathogen response and abiotic stress response.

Another conspicuous module in this tissue is the tan module with ∼4.7% genes up-regulated and ∼8.6% down-regulated. Carbohydrate metabolic processes are down-regulated in panicle as evident from the down-regulated glycosyl hydrolases, glycosyl transferase, sucrose synthase, and β-galactosidase. An important gene GASR7 – Gibberellin-regulated GASA precursor protein is down-regulated. It has been identified as a candidate gene in determining the grain length in rice (Huang et al., 2012) suggesting smaller grain size yield in drought conditions. In the red module, ∼9% of the genes are up-regulated in the panicle tissue and are mostly associated with seed maturation, LEA proteins, dehydrins (involved in abiotic stress) and protein phosphatase 2Cs (involved in hormonal signaling). The midnightblue module has ∼9.7% of the genes up-regulated in the panicle tissue which include stress-induced transcription factors AP2-EREBP, bZIP (involved in hormone signaling) and HSF and other nucleotide binding proteins (ABC transporter, AAA-type ATPase, and ATP-dependent protease La). These nucleotide binding proteins are associated with chaperone like activities, ATP-hydrolysis and proteolytic activities during drought stress.

## Discussion

In the past, several methods have successfully used the concept of ‘guilt by association’ approach to transfer annotations among genes based on certain features such as sequence similarity, similarity in mRNA expression profiles, common biological processes, sharing of protein domains, genes part of the same protein complex or genes which co-regulate or co-evolve (Vandepoele et al., 2009; Ficklin and Feltus, 2011; Lee et al., 2011; Wong et al., 2014). In rice, with only ∼1% of the protein coding genes having experimental evidence for their functions, researchers are increasingly turning to integrated solutions which link the genomic, transcriptomic, proteomic, and metabolomic information under different experimental conditions to understand the plant response mechanisms to abiotic and biotic stress tolerance, cell wall biology, photosynthesis, hormone regulations, immune response, etc. This requires planned experimental studies carried out under different conditions such as tissues, developmental stages or environmental conditions. Here we consider one such experimental study wherein expression profiles in different tissues and developmental stages are obtained under drought stress. The objective of the study is to identify and annotate stress-induced genes that lack functional annotation. Here the co-expressed gene clusters are analyzed using topological based approach for identifying tightly coupled, DEGs that show prevalence of some stress regulatory *cis*-element or are co-regulated by a common transcription factor.

Understanding the molecular mechanisms in drought response can be challenging due to the presence of a large number of complex interactions between 100s of DEGs. Dissecting these complex interactions into a modular view in a tissue- or stage-specific manner can provide a systems-level understanding of drought response. As a first step toward identifying drought stress-induced genes, we construct a network of co-expressed gene modules to first identify drought-responsive modules. This is carried out by analyzing the percentage of DEGs in various tissues and developmental stages and mapping them on to each module. We observe that the two modules, red and midnightblue, exhibit a large fraction of DEGs across all the developmental stages in the three tissues, root, leaf, and panicle. These modules consist of a high percentage of genes, ∼45% (red) and ∼29% (midnightblue), respectively, involved in metabolism. This is in agreement with the emerging view that stress adaptive signaling is tightly linked to the cellular primary metabolism, energy supply and developmental processes. It is observed that many of the top ranked genes based on degree centrality in these two modules also exhibit a high positive fold-change in all the tissues. Hence, for the functional annotation, we select candidate ‘uncharacterized’ genes that are high-degree nodes (top 20%) and up-regulated (fourfold) across all tissues and developmental stages.

Conserved co-expression patterns in functional networks across species provide an effective way to transfer annotations from a model organism to the organism of interest. With the availability of large amount of high-throughput data, a number of such systems-based resources are now available, *viz.*, AraNet (Lee et al., 2014), PlaNet (Mutwil et al., 2011), MetNet Online (Sucaet et al., 2012), etc. First step in any functional annotation transfer is homology-based analysis. In the analysis of red module, we observed that homologs of the uncharacterized rice genes in other plant organisms are reported as “conserved,” “expressed” proteins, i.e., they lack functional annotation in other species as well. So we next analyze the conserved co-expression patterns of homologous genes in the model plant, *Arabidopsis thaliana*. A direct advantage of such an analysis is the elimination of irrelevant gene connections arising in the network due to noise. The subnetwork of the homologs of 13 uncharacterized rice genes is extracted from the *Arabidopsis* co-function network in AraNet and mapped on to the red module. This resulted in a network of 40 genes (13 uncharacterized genes and its 27 conserved neighbors), shown in **Figure 3**. Analysis of such conserved subnetworks provides reliable extrapolation of functional annotation to the uncharacterized genes from their conserved neighborhood. We observe terms such as “seed development,” “response to ABA,” and “water deprivation” as a common theme among the closely connected genes in this subnetwork. Motif analysis of this subnetwork genes show that they all share ABREs and auxin-associated *cis*-element motifs in their promoter sequences. It is known that majority of ABA-regulated genes share the conserved ABRE motif. In a study by Seo and Park (2009), it has been reported that ABA and auxin play critical roles in root growth under drought through complex signaling networks, suggesting a strong relationship between the two phytohormones, ABA, and auxin. Thus, by the above approach, we are able to show that 13 previously uncharacterized genes are involved in ABA and auxin mediated signaling pathways.

Midnightblue module is observed to be another important drought-responsive module across all stages and tissues, especially root and leaf, with the top GO term indicating nuclease binding activities. High degree and up-regulated genes in this module include many stress responsive genes, namely, those involved in nucleobase-containing compound metabolic processes including ATP binding, suggesting a role in the regulation of ATP synthesis and transport as well as proteolytic activities which are significant during drought. Combining the information from AraNet and analysis of *cis*-regulatory elements, we predict that the three uncharacterized genes in this module are also induced in an ABA-dependent manner. The association of these ABA regulated genes of transcription factors along with other high degree and up-regulated genes such as bZIP (*LOC_Os01g64730*) and HSF (*LOC_Os06g35960*) transcription factors, stress-responsive genes such as AAA-type ATPase, ATP-dependent protease, etc., suggest a possible cross-talk in the proteolytic activities during drought.

We observed that the green module displayed a tissue-specific response in leaves, especially in the panicle elongation stage. A number of up-regulated genes involved in small molecule metabolic processes, protein amino acid phosphorylation as well as regulation of gene expression (transcription factors) are observed in this module. It is interesting to observe that the blue and yellow modules, having a role in photosynthesis and associated metabolic processes, are down-regulated and this effect is more profound in the panicle elongation stage (**Figure 2**). The growth and morphology of the panicle is a key factor in determining the yield (Li et al., 2010). A reduction of photosynthetic activities during this stage suggests that the grain yield may be affected due to drought.

An alternate approach to the identification of tissue-specific, drought-responsive genes is discussed in the analysis of the green module. Considering transcription factor as a guide-gene, a subnetwork of co-regulated genes is obtained. A number of studies (Mao et al., 2009; Fu and Xue, 2010; Wong et al., 2014) have used this approach where ‘bait genes’ with known functions are used in querying the co-expression network. The resulting subnetwork consists of the guide gene along with its first neighbors associated with each other possibly due to a common biological process. Here we consider OsMYB2 transcription factor as guide-gene and construct its subnetwork by considering differentially expressed, tightly coupled first neighbors. OsMYB2 does not have a characterized ortholog in the other plant species. However, functional analysis of its first-neighbors shows their involvement in various biotic and abiotic stress responses. A few of its neighbors in the subnetwork are uncharacterized. Promoter analysis of these sequences confirmed the presence of MYB binding sites in the uncharacterized genes and other up-regulated neighboring genes. In plants, MYB transcription factors are known to be involved in key processes such as development, secondary metabolism hormone signaling, disease resistance and abiotic stress response (Allan et al., 2008; Cominelli and Tonelli, 2009). In the promoter regions of the uncharacterized genes, a number of ABRE motifs were also detected. Several studies have indicated the accumulation of ABA in vegetative tissues during drought and stomatal closure is one of its key functions in leaves (Trejo et al., 1993; Xiong and Zhu, 2003).

## Conclusion

In this study, we present a co-expression network-based approach for functional annotation of uncharacterized genes in rice under drought stress. The study reveals the role of topological properties of gene co-expression networks to identify drought-responsive modules in a tissue-specific manner. Here we consider clusters of co-expressed genes/transcription factors that are well-connected, have a conserved neighborhood across species and share common *cis*-elements. Analysis of such clusters provides a powerful approach for the functional annotation of genes in response to environmental stress. By this approach, our attempt to provide functional annotation to 13 uncharacterized genes in the red module indicates their involvement in ABA and auxin mediated signaling pathways and suggests a cross-talk between ABA-regulated and auxin-responsive genes in response to drought stress. Similarly, the functional annotation of three uncharacterized genes in midnightblue module suggest they are activated in an ABA-dependent manner and their association with other transcription factors and protease genes suggest a possible cross-talk in the proteolytic activities during drought. Alternatively, in the situation when homologs of a transcription factor and its first degree neighbors are not present in model plant organism, we proposed single guide-gene approach. Based on this analysis, the 10 uncharacterized neighbors of OsMYB2 transcription factor are shown to be associated with ABA-response, leaf desiccation and photosynthesis. The proposed approach is particularly useful when the genes lack a known domain or functionally characterized homologs in the database. We expect that on integrating other types of information, such as protein–protein interaction, phylogeny and RNAseq data may result in reliable function predictions.

## Conflict of Interest Statement

The authors declare that the research was conducted in the absence of any commercial or financial relationships that could be construed as a potential conflict of interest.
