# Supplementary material for: Functional characterization of drought-responsive modules and genes in Oryza sativa: a network-based approach
Source: Front Genet. 2015 Jul 30;6:256. doi: 10.3389/fgene.2015.00256 (PMC4519691; doi:10.3389/fgene.2015.00256)
Supplement: Supplementary file 1 [file Supplementary_Material.DOCX]

***Supplementary Material***

**Functional characterization of Drought-responsive Modules and Genes in *Oryza sativa*: A Network-based Approach**

**Sanchari Sircar and Nita Parekh***

*** Correspondence:** Nita Parekh: [nita@iiit.ac.in](mailto:nita@iiit.ac.in)

## Supplementary Tables

**Module quality statistics:**

To assess the preservation or robustness of co-expressed gene modules in the network constructed in this study, we use the network module preservation statistics implemented in the function *modulePreservation* in the WGCNA R package. Network module preservation statistics assess whether the density and connectivity patterns of modules defined in a reference data set are statistically significant compared to a set of randomly clustered genes. We carried out a permutation test in which gene labels are randomly permuted 200 times, and various module quality statistics (*viz*., density-based, connectivity-based, and separability based preservation statistics) are computed. For each statistic, Z-score is computed which provides evidence that a module is preserved more significantly than a random sample of all network genes, and *p*-value which gives the probability of seeing the module quality statistic in a random sample of genes of the same size, respectively. The results are summarized as *Zsummary* and log *psummary* for various statistics: (1) Proportion of variance explained by eigengenes (*propVarExplained*), (2) eigengene based mean connectivity, or module membership (*meanSignAwareKME*), (3) mean intramodular adjacency (*meanAdj*), (4) maximum adjacency ratio (*meanMAR*), and (5) mean correlation density (*meanSignAwareCorDat*). A Z-score > 10 gives a strong evidence for network connectivity preservation and robustness of the modules (Langfelder et al., 2011). In Table S1, all the modules have a significant Z-score with a very low *psummary* (~ 0.0), except the gold and grey modules. The grey module consists of genes which were not clustered into any module in our network, while the gold module consists of 1000 randomly selected genes that represent a sample of the whole network, constructed for the module preservation analysis.

**Table S1: Module quality statistics computed for the 16 co-expressed modules in our network. The results are summarized for various network module quality statistics as *Zsummary* and log *psummary* providing evidence for the robustness of these modules.**

| **S. No.** | **Modules** | **Size** | **Zsummary.qual** | **log (psummary.qual)** |
| --- | --- | --- | --- | --- |
| 1 | Black | 1000 | 63.70 | -9.11E+02 |
| 2 | Blue | 1000 | 89.43 | -1.87E+03 |
| 3 | Brown | 1000 | 65.04 | -9.69E+02 |
| 4 | Cyan | 456 | 60.09 | -8.46E+02 |
| 5 | Green | 1000 | 72.50 | -1.23E+03 |
| 6 | Greenyellow | 655 | 42.86 | -4.10E+02 |
| 7 | Lightcyan | 296 | 47.87 | -5.31E+02 |
| 8 | Magenta | 724 | 66.70 | -1.02E+03 |
| 9 | Midnightblue | 385 | 37.96 | -3.21E+02 |
| 10 | Pink | 809 | 82.66 | -1.61E+03 |
| 11 | Purple | 716 | 71.06 | -1.18E+03 |
| 12 | Red | 1000 | 62.41 | -8.81E+02 |
| 13 | Salmon | 605 | 44.71 | -4.43E+02 |
| 14 | Tan | 614 | 57.34 | -7.69E+02 |
| 15 | Turquoise | 1000 | 82.09 | -1.52E+03 |
| 16 | Yellow | 1000 | 56.55 | -7.17E+02 |
| 17 | Gold | 1000 | 0.212 | -3.87E-01 |
| 18 | Grey | 353 | -9.01 | -1.86E-15 |

**Alternative Network Inference Methods:**

To provide biological significance to gene-pair associations in the conserved clusters in red and green modules used for functional extrapolation, two different network constructions, *viz*., co-expression networks and context likelihood of relatedness (CLR) networks were constructed, considering various association rules for gene-pair relatedness.

**Correlation-based networks:** Following association methods were used for network construction:

- **Pearson Correlation:** It measures the strength of linear relationship between two random variables *x* and *y* with *n* measurements and standard deviations S_x_ and S_y_, and is defined as:

$$\boldsymbol{r}=\frac{\sum_{i=1}^{n} (x_{i-}\bar{x}) (y_{i-}\bar{y})}{(n-1)S_{x}S_{y}}$$

- **Spearman’s Rank Correlation:** It is a non-parametric measure and measures the nonlinear monotonic relationship between two variables by linear relationship between the ranks of the values of the two variables and is given by:

$$\boldsymbol{r}_{\boldsymbol{s}}=\frac{6\sum_{i=1}^{n} d_{i}^{2}}{n(n^{2}-1)}$$

where *d*_i_= difference in paired ranks and n = number of cases.

**Context likelihood of relatedness (CLR):** It is an extension of the relevance networks class of algorithms for identifying transcriptional regulatory interactions (Faith et al. 2007). It uses mutual information for scoring the similarity between the expression levels of two genes. A gene and a transcription factor are predicted to interact if the mutual information between the expression levels of the gene and its potential regulator is above some set threshold (background distribution of the MI scores). The following association methods were used for network construction in this case.

- **Mutual Information (MI):** Given two random variables X, Y , with respective ranges x_i_ ∈ A_i_, y_j_ ∈ A_j_ and probability distributions functions P(X = x_i_) ≡ p_i_, P(Y = y_j_) ≡ p_j_, the Mutual Information (I) between two random variables is defined as:

$$I\left( X,Y \right)= \sum_{i} \sum_{j} p_{ij}log\frac{p_{ij}}{p_{i}p_{j}}$$

where *p_ij_* is the joint probability distribution function of *X* and *Y*, and p_i_ and p_j_ are the marginal probability distribution functions of *X* and *Y* respectively.

- **Maximal Information Coefficient (MIC):** It belongs to a larger class of maximal information-based nonparametric exploration statistics for identifying and classifying relationships. It uses binning as a means to apply mutual information on continuous random variables such that that the mutual information between the variables is maximal over the bins (Reshef et al. 2011).

**Table S2: For assessing the significance of gene-pair associations, four networks were constructed and analyzed. The parameters used and other details are summarized.**

| **Network Type** | **Association Estimation Method** | **Association Cutoff** | **No of genes in overall network** | **MCL (inflation parameter)** | **No. of Modules** |
| --- | --- | --- | --- | --- | --- |
| Co-expression | Spearman’s rank Correlation | 0.6 | 18732 | 1.2 | 6 |
|  | Pearson Correlation | 0.6 | 18642 | 1.2 | 10 |
| CLR-based | Mutual Information (MI) | 3.8 | 18796 | 1.4 | 15 |
|  | Maximal information coefficient(MIC) | 3.8 | 18790 | 1.3 | 17 |

**Table S3: Comparison of conserved gene-pair associations in WGCNA and four alternate networks for two gene-clusters. UGs - uncharacterized genes, CGs - characterized genes.**

| **Network Type** | **Association Estimation Method** | **No. of Genes (Nodes) in** | | **Edges between 13 UGs and 27 CGs in Red-AraNet cluster** | **Edges between OsMyb2 and 47 neighbors** |
| --- | --- | --- | --- | --- | --- |
|  |  | **Red-AraNet cluster** | **MyB cluster** |  |  |
| Correlation | WGCNA | 40 | 48 | 346 | 47 |
|  | Spearman | All 40 in the same cluster | 47: cluster C1  1: cluster C2 | 329 | 46 |
|  | Pearson | 37: cluster C2  2: in C1 | All 48 in the same cluster | 297 | 47 |
| CLR-based | Mutual Information (MI) | All 40 in the same cluster | All 48 in the same cluster | 293 | 46 |
|  | Maximal information coefficient (MIC) | 37: cluster C4  3: in each of cluster C1, C2 & C5 | 45: cluster C1  3: cluster C3 | 140 | 32 |

## Supplementary Figures

The Rice Genome Annotation Database (RGAP) (Kawahara et al. 2013), apart from providing annotations for the rice genome, also provides gene co-expression information from a number of rice datasets across varying experimental conditions and tissues. To confirm the co-expression profiles of the 13 uncharacterized genes and their 27 conserved neighbors, we searched against the abiotic stress-associated dataset GSE6901 in RGAP, (GSE6901 – transcription profiles from a seven-day-old rice seedlings grown in the presence of light, under control and stress conditions: drought, cold, and salinity). We observed that 10 out of 13 uncharacterized genes and 11 out of 27 annotated genes are also co-expressed (as shown by expression profiles of these 21 genes in Figure S1) and belong to the same module (turquoise) in this experimental study. The GSE6901-turquoise module is shown to be associated with genes differentially expressed due to drought and salt stress by Childs et al. (2011).


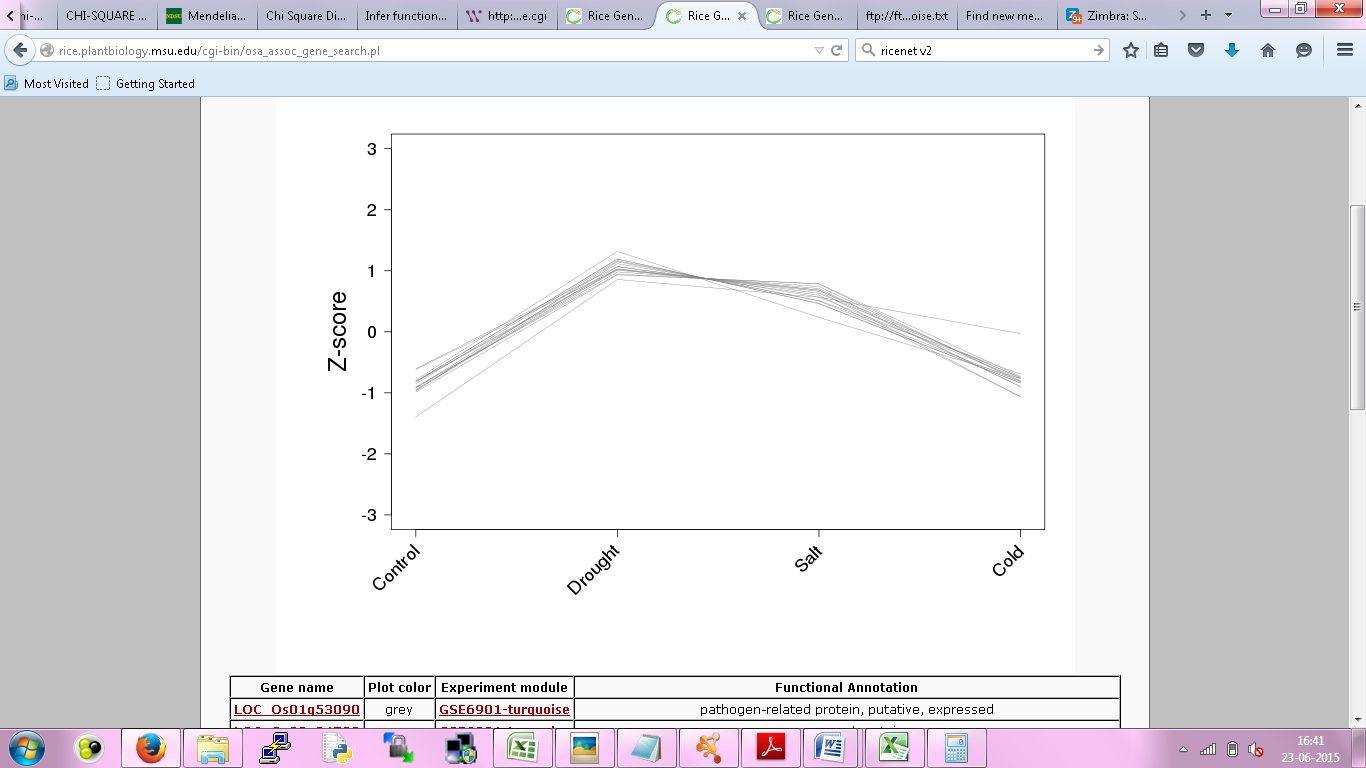


**Figure S1: Normalized expression profiles of 10 out of 13 uncharacterized genes, and 11 out of 27 annotated genes from GSE690 experiment study in RGAP shows a good correlation between these genes.**

**References:**

Childs, K. L., Davidson, R. M., & Buell, C. R. (2011). Gene coexpression network analysis as a source of functional annotation for rice genes. *PloS One*, *6*(7), e22196.

doi:10.1371/journal.pone.0022196

Faith JJ, Hayete B, Thaden JT, Mogno I, Wierzbowski J, Cottarel G, Kasif S, Collins JJ, Gardner TS. 2007. Large-scale mapping and validation of Escherichia coli transcriptional regulation from a compendium of expression profiles. *PLoS biology*, 5(1), e8. doi:10.1371/journal.pbio.0050008

Kawahara, Y., de la Bastide, M., Hamilton, J. P., Kanamori, H., McCombie, W. R., Ouyang, S., … Matsumoto, T. (2013). Improvement of the Oryza sativa Nipponbare reference genome using next generation sequence and optical map data. *Rice (New York, N.Y.)*, *6*(1), 4. doi:10.1186/1939-8433-6-4

Langfelder, P., Luo, R., Oldham, M. C., & Horvath, S. (2011). Is my network module preserved and reproducible? *PLoS Computational Biology*, *7*(1), e1001057. doi:10.1371/journal.pcbi.1001057

Reshef DN, Reshef YA, Finucane HK, Grossman SR, McVean G, Turnbaugh PJ, Lander ES, Mitzenmacher M, Sabeti PC. 2011. Detecting novel associations in large data sets. *Science* 334:1518–24. doi: 10.1126/science.1205438
